# Room-temperature NO_2_ sensing performance of ZnO/Ti_3_C_2_T*_x_* heterojunctions

**DOI:** 10.3762/bjnano.17.79

**Published:** 2026-08-19

**Authors:** Ngo Thi Chau, Quoc Vinh Ho, Dai Hai Nguyen, Phan Khanh Thinh Nguyen, Qui Thanh Hoai Ta

**Affiliations:** 1 Institute of Advanced Technology, Vietnam Academy of Science and Technology, 1B TL29 Street, An Phu Dong Ward, Ho Chi Minh City 700000, Vietnam; 2 Graduate University of Science and Technology, Vietnam Academy of Science and Technology, 18 Hoang Quoc Viet Street, Hanoi 100000, Vietnam; 3 Faculty of Pharmacy, Nguyen Tat Thanh University, 300A Nguyen Tat Thanh Street, Ho Chi Minh City 700000, Vietnam; 4 School of Chemical, Biological, and Battery Engineering, Gachon University, 1342 Seongnamdaero, Sujeong-gu, Seongnam-si, Gyeonggi-do, 13120, Republic of Korea

**Keywords:** gas sensor, room temperature, ZnO/Ti_3_C_2_T*_x_* composites

## Abstract

In recent years, metallic Ti_3_C_2_T*_x_* MXenes have garnered considerable attention in the realm of gas sensors owing to their distinctive characteristics, including high conductivity, inherent hydrophilicity, and abundant surface termination groups. Nonetheless, Ti_3_C_2_T*_x_*-based sensing composites encounter challenges related to response/recovery time and low sensitivity, thereby limiting their applicability across diverse environmental conditions. Addressing these limitations, we present the synthesis of ZnO/Ti_3_C_2_T*_x_* nanocomposites via a facile method for gas sensing applications. The optimized composite exhibits a notable response of around 6.1% to 5 ppm NO_2_, coupled with remarkable selectivity at ambient temperatures. Moreover, the sensor demonstrates exceptional reproducibility across multiple testing iterations. The observed enhancement in gas sensing performance is attributed to the abundance of oxygen vacancies and surface functional groups within the ZnO/Ti_3_C_2_T*_x_* composites, which facilitate robust interactions with NO_2_ molecules. These findings underscore the efficacy of ZnO/Ti_3_C_2_T*_x_* nanocomposites as a viable strategy for enhancing the gas sensing properties of Ti_3_C_2_T*_x_*-based sensors.

## Introduction

Nowadays, increasing attention is paid to the early detection and prevention of chemical leaks and accidental releases, highlighting the importance of identifying trace-level chemicals in gaseous emissions [1–3]. Notably, a significant subset of such accidents involves the leakage of toxic gases and combustible substances, including carbon monoxide (CO), sulfur dioxide (SO_2_), carbon dioxide (CO_2_), ammonia (NH_3_), and nitrogen dioxide (NO_2_) [4–6]. Of these, NO_2_ stands out as a ubiquitous emission arising from daily activities and vehicular operations, primarily attributed to fossil fuel combustion and engine emissions. Instances of NO_2_ exposure at low concentrations (≈10 ppm) have been associated with adverse health effects, including nasal and ocular irritation as well as throat discomfort [7–8]. Prolonged exposure to elevated NO_2_ concentrations can bring about severe respiratory ailments such as pulmonary edema and asthma, with fatal outcomes observed at concentrations exceeding 100 ppm. Furthermore, the release of NO_2_ into the natural environment poses a significant risk, potentially leading to soil and water contamination through acid rain deposition [9–11].

In recent decades, research on two-dimensional (2D) layered materials has grown significantly owing to their unique physicochemical properties. Materials such as graphene, black phosphorus, transition metal dichalcogenides, and MXenes have attracted considerable attention for a wide range of applications [12–14]. MXenes have emerged as particularly promising candidates in the realm of gas sensing owing to their distinctive attributes [15]. The discovery of Ti_3_C_2_T*_x_* MXene in 2011, achieved through the selective etching of Ti_3_AlC_2_ using LiF and HCl, marked a pivotal milestone in the exploration of MXene-based gas sensors [16–17]. Ti_3_C_2_T*_x_* MXene has exhibited commendable sensor performance at ambient temperatures, characterized by a low signal-to-noise ratio and high electron conductivity, rendering it a subject of extensive investigation in sensing applications. Nonetheless, the inherent limitations of MXenes, including suboptimal sensitivity and long recovery times, necessitate remediation prior to broader application [18–19].

In particular, the amalgamation of Ti_3_C_2_T*_x_* MXene with metal oxide semiconductors has garnered considerable attention as a strategy to augment the NO_2_ sensing capabilities of Ti_3_C_2_T*_x_*-based gas sensors, attributed to the formation of Schottky barriers and the increase of surface area [20–21]. For instance, Choi et al. synthesized a TiO_2_@Ti_3_C_2_T*_x_* composite utilizing an in situ oxidation technique and modulating oxidation parameters to regulate the formation of Schottky barriers; the composite exhibited superior NO_2_ responsiveness compared to pristine Ti_3_C_2_T*_x_* MXene [22]. Ta et al. reported promising results with a heterostructure comprising Si and MXene-derived TiO_2_, demonstrating robust NO_2_ sensing performance [23]. Similarly, Sun et al. employed Co_3_O_4_ nanoparticle-based polyethyleneimine-functionalized Ti_3_C_2_T*_x_* nanosheets, showcasing exemplary sensing response characteristics and rapid response/recovery times towards NO_2_ gas [24].

Furthermore, n-type ZnO has garnered significant traction as a NO_2_ sensor owing to its wide bandgap energy (3.37 eV), abundant oxygen vacancies, and exceptional chemical stability [25–26]. Moon et al. [27] synthesized a rGO/ZnO composite for NO_2_ detection at room temperature, while Song et al. [28] employed a hydrothermal approach to fabricate ZnO nanofibers, yielding enhanced NO_2_ sensing performance. Notably, ultraviolet (UV) irradiation has emerged as an efficacious strategy to enhance the recovery kinetics of gas-sensitive materials by facilitating desorption reactions. Zhang et al. demonstrated the efficacy of TiO_2_/SnO_2_ under UV illumination, manifesting superior response/recovery kinetics compared to sensors based solely on TiO_2_ nanostructures [9]. In other words, the synergistic integration of Ti_3_C_2_T*_x_* MXene with metal oxide semiconductors, alongside innovative strategies such as combination, Schottky barriers, and UV irradiation, holds promise for advancing the development of robust and efficient NO_2_ sensing platforms, thereby contributing to enhanced environmental monitoring and public health safeguards [29–31]. Further research endeavors aimed at refining sensor materials and elucidating underlying mechanisms are warranted to realize the full potential of these novel sensing architectures in real-world applications. Motivated by the promising outcomes observed with n-type ZnO nanomaterials, the integration of Ti_3_C_2_T*_x_* MXene and ZnO offers a compelling avenue to capitalize on the synergistic effects of Schottky junction formation, leveraging the distinctive sensing properties inherent in both components to enhance NO_2_ sensing performance.

In this work, we describe a simple synthesis approach for fabricating ZnO/Ti_3_C_2_T*_x_* composite materials, involving the physical combination of ZnO nanostructures and Ti_3_C_2_T*_x_* MXene. This simplified technique serves to mitigate the oxidation tendencies of MXene while promoting the formation of interactions with ZnO nanorods. The optimized heterostructures exhibit enhanced NO_2_ sensing activity at ambient temperatures, presenting a viable strategy for advancing gas sensing technologies.

## Results and Discussion

### Crystal structural and morphological studies

**Figure 1:**
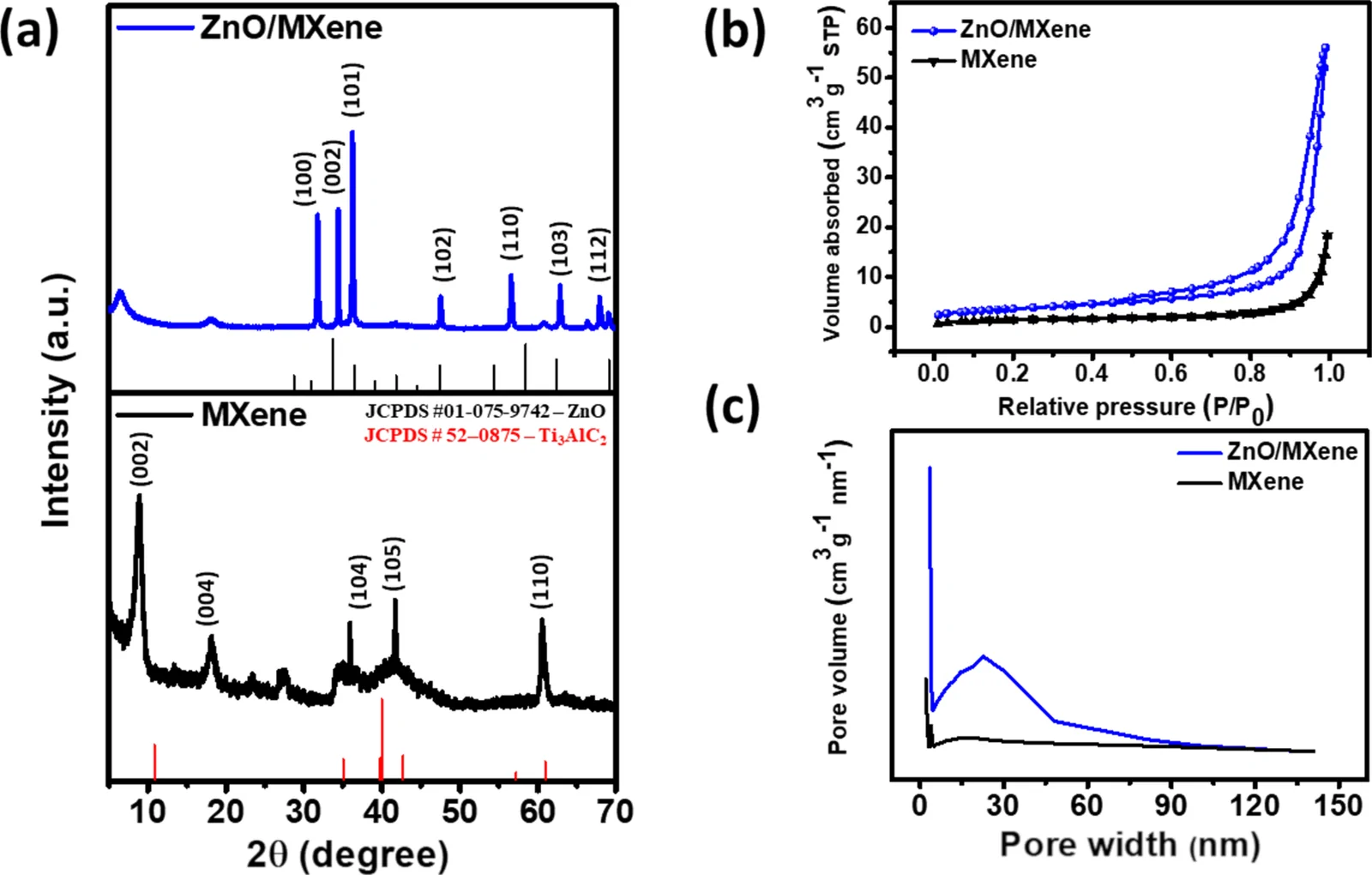
(a) XRD patterns, (b) nitrogen adsorption/desorption isotherms, and (c) pore-size distributions of pure Ti_3_C_2_T*_x_* MXene and ZnO/Ti_3_C_2_T*_x_* composites.

Figure 1a displays the XRD patterns of pure Ti_3_C_2_T*_x_* MXene and ZnO/Ti_3_C_2_T*_x_* composite. The XRD pattern of pristine Ti_3_C_2_T*_x_* MXene exhibits a strong characteristic (002) reflection at 2θ = 8.9°, together with weaker diffraction peaks at approximately 18.2°, 36.0°, 41.1°, and 60.8°, corresponding to the (004), (104), (105), and (110) planes of Ti_3_C_2_T*_x_*, respectively (JCPDS no. 52-0875). The intense (002) peak, accompanied by the absence of the characteristic Ti_3_AlC_2_ MAX phase reflections, confirms the selective removal of the Al atomic layers during the etching process and the formation of Ti_3_C_2_T*_x_* MXene. For the ZnO/Ti_3_C_2_T*_x_* composite, the prominent diffraction peaks located at 2θ = 31.7°, 34.4°, and 36.2° are indexed to the (100), (002), and (101) planes of hexagonal wurtzite ZnO (JCPDS no. 01-075-9742), while the remaining reflections are also in good agreement with the standard ZnO pattern, confirming the crystallization of ZnO nanostructures [32–34]. Notably, the characteristic (002) peak of Ti_3_C_2_T*_x_* shifts from 8.9° to 6.7° after ZnO loading and becomes broader. According to Bragg’s law, this shift toward a lower diffraction angle corresponds to an increase in the interlayer spacing, suggesting that ZnO nanostructures are effectively intercalated between adjacent MXene nanosheets. The enlarged interlayer distance suppresses the restacking of Ti_3_C_2_T*_x_* layers, exposes more accessible active sites, and facilitates charge transport and ion diffusion within the composite structure. Moreover, no additional diffraction peaks associated with impurity phases or reaction by-products are detected, demonstrating the phase purity of the synthesized ZnO/Ti_3_C_2_T*_x_* composite.

The pore size distribution and specific surface area (SSA) of selected samples were further determined using nitrogen adsorption/desorption, as shown in Figure 1b. The plots illustrate a type-IV isotherm at relative pressures from 0.6 to 1.0 with a hysteresis cycle. The SSA of the composite analyzed by the BET method is 12.8 m^2^/g, which is more than twice that of pure Ti_3_C_2_T*_x_* (5.1 m^2^/g). This substantial enhancement in SSA can be attributed to the introduction of ZnO nanostructures within the MXene layers as well as to synergistic effects of the composite constituents, which provide more active sites for interaction with gas molecules. The pore size distribution of the composite ranged from approximately 2 to 120 nm, ascribing a hierarchical porous structure composed of both mesopores (2–50 nm) and macropores (>50 nm) (Figure 1c).

**Figure 2:**
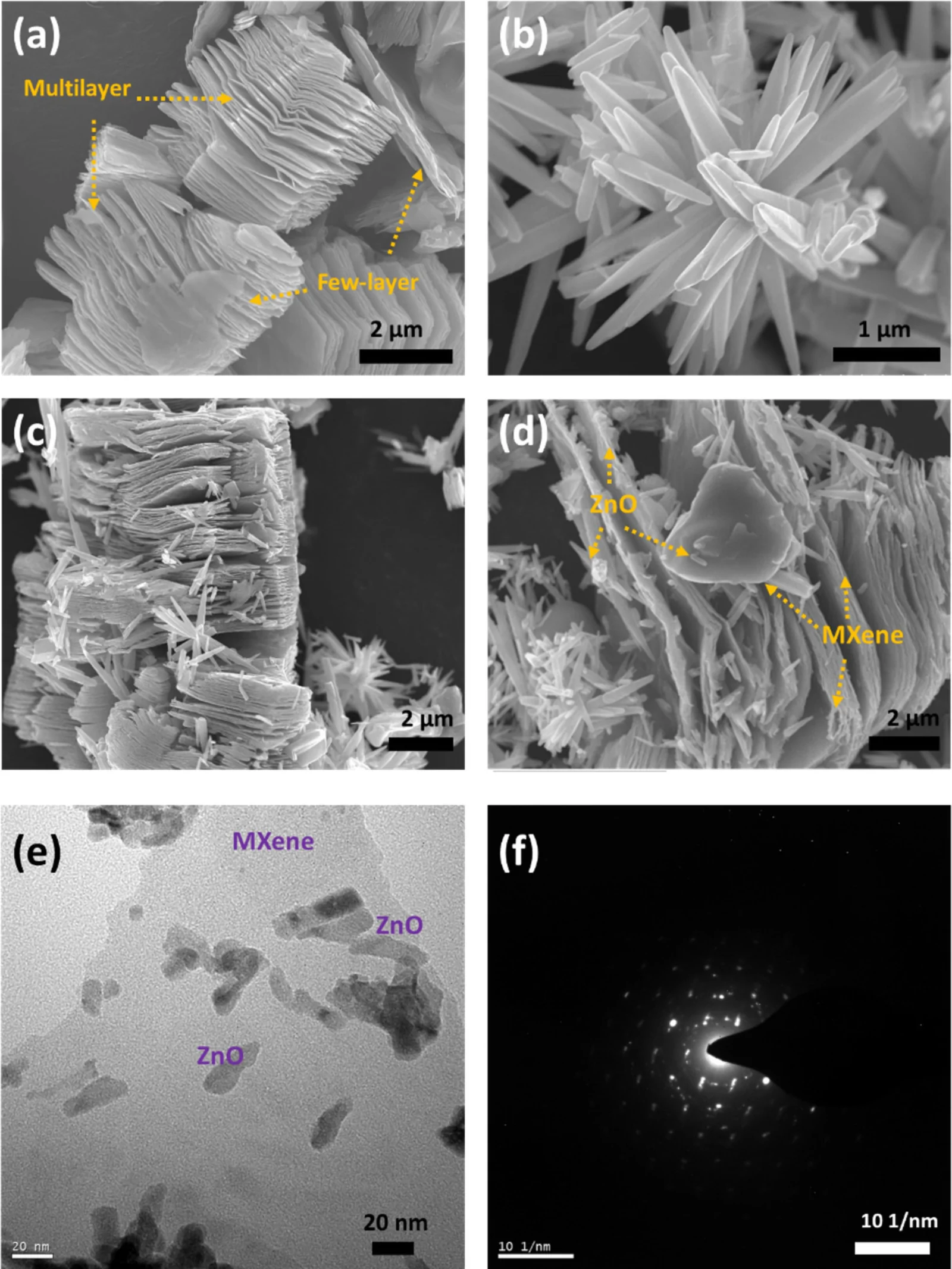
FESEM images of (a) pure Ti_3_C_2_T*_x_* MXene, (b) pristine ZnO, and (c, d) ZnO/Ti_3_C_2_T*_x_* composites. (e) TEM image and (f) corresponding selected-area electron diffraction pattern of ZnO/Ti_3_C_2_T*_x_* composites.

Figure 2 displays FESEM images of the pure Ti_3_C_2_T*_x_* MXene, pristine ZnO nanostructures, and ZnO/Ti_3_C_2_T*_x_* composites. The pure MXene shows a characteristic accordion-like morphology, in which both few-layered nanosheets and multilayered stacked lamellae with enlarged interlayer spacing were observed, which is typical of etched MXene materials (Figure 2a). ZnO prepared by the chemical bath deposition method displays a flower-like morphology consisting of radially assembled rod-like nanostructures (Figure 2b). After composite formation, the ZnO/Ti_3_C_2_T*_x_* composite retains the structure of MXene, while ZnO nanostructures are randomly anchored onto the surface and interlayer spaces of the MXene sheets (Figure 2c,d). Such an intimate interfacial contact is expected to facilitate efficient charge transfer and provide abundant active sites for gas sensing. The TEM image further confirms the decoration of ZnO nanostructures on a locally exfoliated Ti_3_C_2_T*_x_* MXene nanosheet, indicating that partial exfoliation of the MXene occurred during sample preparation. Moreover, the corresponding selected-area electron diffraction pattern displays distinct diffraction rings, indicating the crystalline nature of ZnO/Ti_3_C_2_T*_x_* composite (Figure 2e,f).

**Figure 3:**
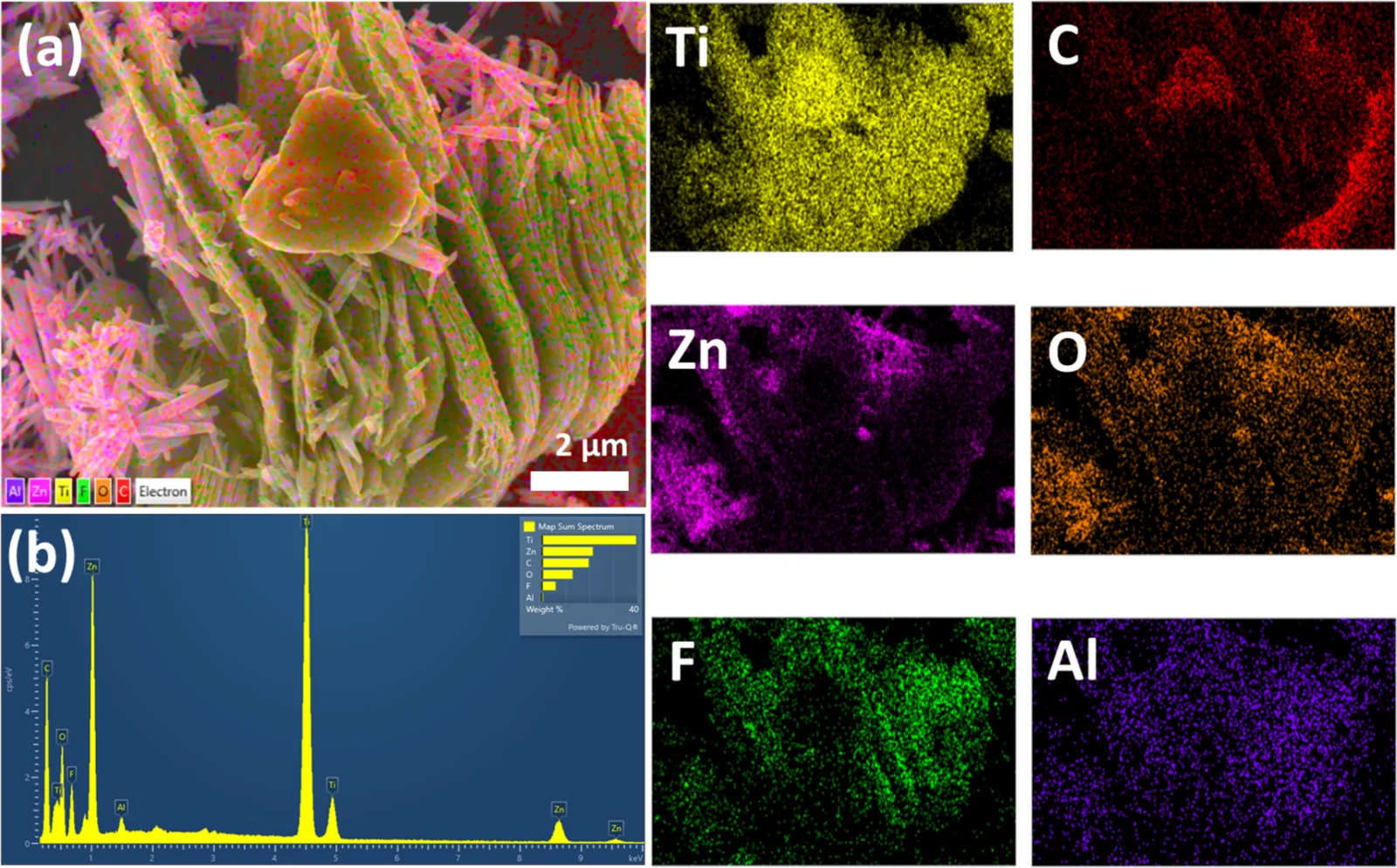
(a) Elemental mapping images, (b) EDS images of ZnO/Ti_3_C_2_T*_x_* composites.

In the hierarchical structure, ZnO plays an important role as a catalytic component. We analyzed the structure of ZnO/Ti_3_C_2_T*_x_* using SEM-EDS mapping to observe the decoration of ZnO relative to Ti_3_C_2_T*_x_* MXene. As shown in Figure 3, the elemental mapping of the composite shows that Ti_3_C_2_T*_x_* nanosheets constitute the framework, and ZnO nanostructures were finely decorated throughout the bulk system. The elements Zn, O, Ti, and C appear in the spectra, which further confirms that ZnO nanostructures are effectively contained in the composite.

### Optical properties

**Figure 4:**
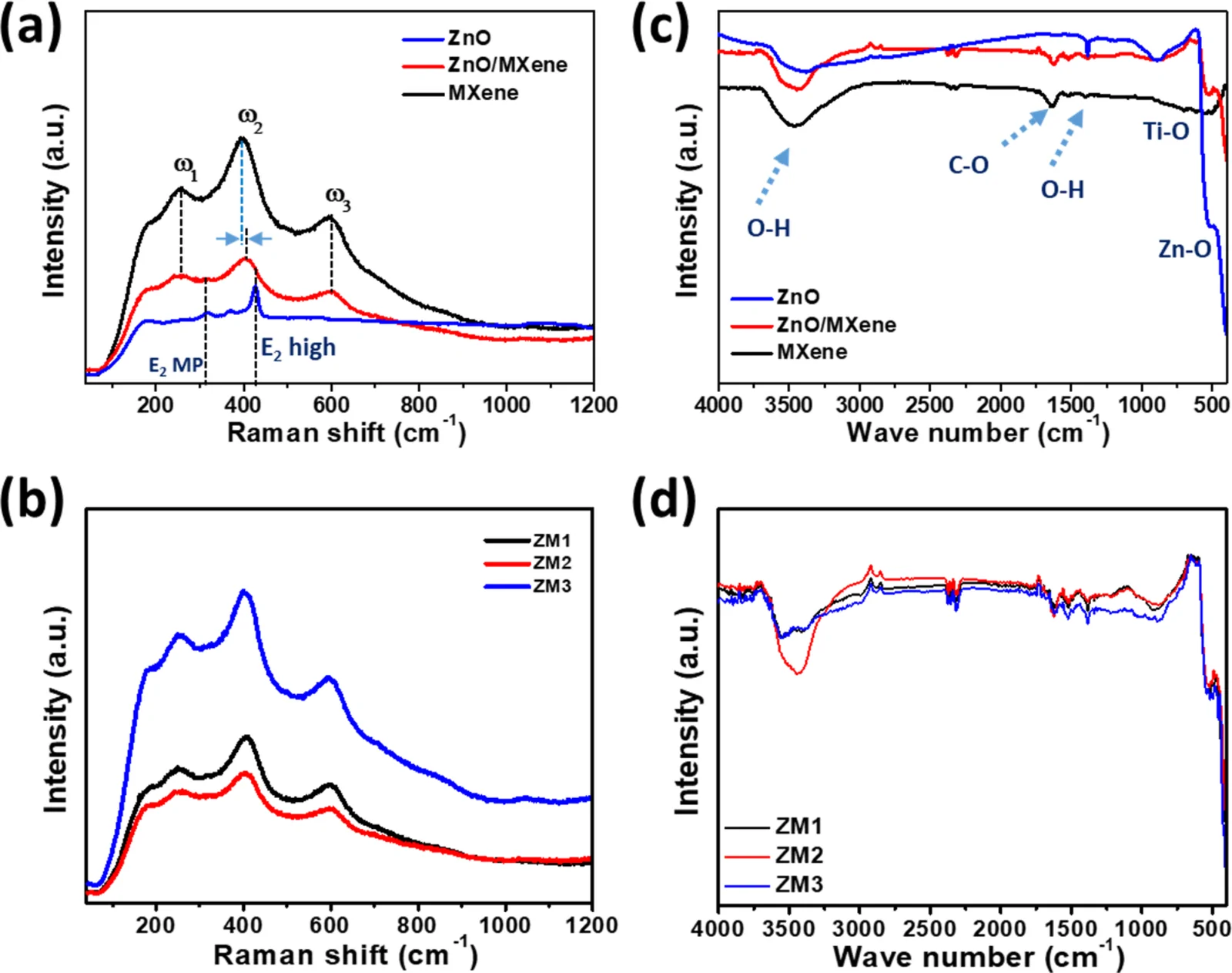
(a,b) Raman spectra, and (c,d) FTIR of synthesized samples.

Raman spectroscopy was performed on selected samples to study their characteristic vibrational modes, as shown in Figure 4a,b. For the ZnO sample, strong peak intensities are observed at 327 and 415 cm^−1^, confirming the presence of wurtzite-phase ZnO, with multiple-phonon scattering *E*_g_ and the high-frequency E_2_ mode, respectively [35]. Peaks located around 230 cm^−1^ (ω_1_), 405 cm^−1^ (ω_2_), and 607 cm^−1^ (ω_3_) are attributed to titanium carbide vibrational modes in the ZnO/MXene composite, while the vibrational modes at 327 and 415 cm^−1^ overlap with those of ZnO [36]. Notably, with the introduction of ZnO, the highest Raman signal for MXene in the composite exhibited a slight shift towards higher wavenumbers compared to pure MXene, preliminarily suggesting the formation of Ti–O–Zn and Zn–Ti bonds, which could act as diffusion bridges to facilitate electron transport [37–38].

In addition, FTIR measurements were conducted for the synthesized samples, as shown in Figure 4c,d. The spectrum of pure ZnO exhibits characteristic bands at 400 cm^−1^, corresponding to Zn–O bonds in ZnO, and at 3500 cm^−1^, indicating the stretching vibration of the O–H groups. For Ti_3_C_2_T*_x_*, the presence of Ti–O bending groups with mode A_2u_ (550 cm^−1^), O–H bending (1450 cm^−1^), and O–H stretching (3490 cm^−1^) is observed [39]. After the introduction of ZnO onto the surface of the Ti_3_C_2_T*_x_* MXene composite, all corresponding peaks are clearly visible in the spectra. These results confirm the successful growth of ZnO within the composite.

**Figure 5:**
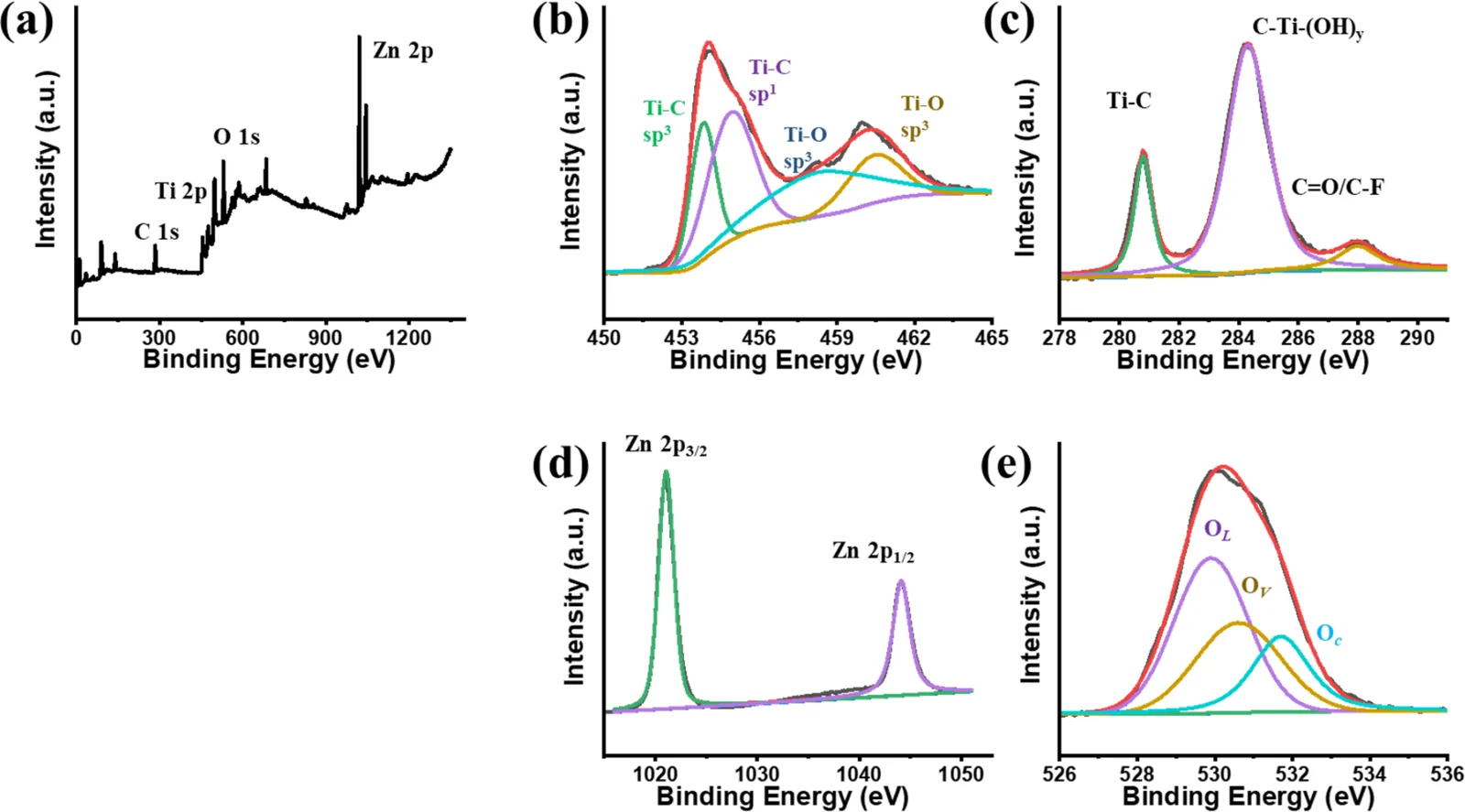
(a) The XPS spectra of selected ZnO/Ti_3_C_2_T*_x_* composite, (b) Ti 2p, (c) C 1s, (d) Zn, (e) O 1s.

The surface chemical states and binding energies of the optimized sample were analyzed using X-ray photoelectron spectroscopy. As shown in Figure 5a, the survey spectrum of ZnO/Ti_3_C_2_T*_x_* confirms the coexistence of Ti, C, Zn, and O on the surface of the composite, consistent with the EDS mapping results. For the Ti 2p region (Figure 5b), the main peak at 454.1 eV is assigned to Ti–C bonds, while the peaks at 458.2 and 460.3 eV correspond to Ti–O bonds, respectively [40–41]. The C 1s spectrum (Figure 5c) displays binding energies of 280.8, 284.2, and 288.1 eV, corresponding to Ti–C, C–Ti–(OH)*_y_*, and C=O/C–F bonds, respectively [42]. The high-resolution Zn 2p spectra (Figure 5d) exhibit spin–orbit doublets at 1021.2 eV (Zn 2p_3/2_) and 1044.1 eV (Zn 2p_1/2_), which are characteristic of Zn^2+^ in ZnO [43]. The O 1s spectrum revealed components attributed to chemisorbed oxygen species (531.6 eV), lattice oxygen (529.8 eV), and oxygen vacancies (530.7 eV), suggesting the presence of abundant hydrophilic surface terminations (T*_x_*) [44] (Figure 5e). These enriched oxygen species are expected to provide active sites for target gas interactions, thereby enhancing the sensor response.

### Gas sensing activities

**Figure 6:**
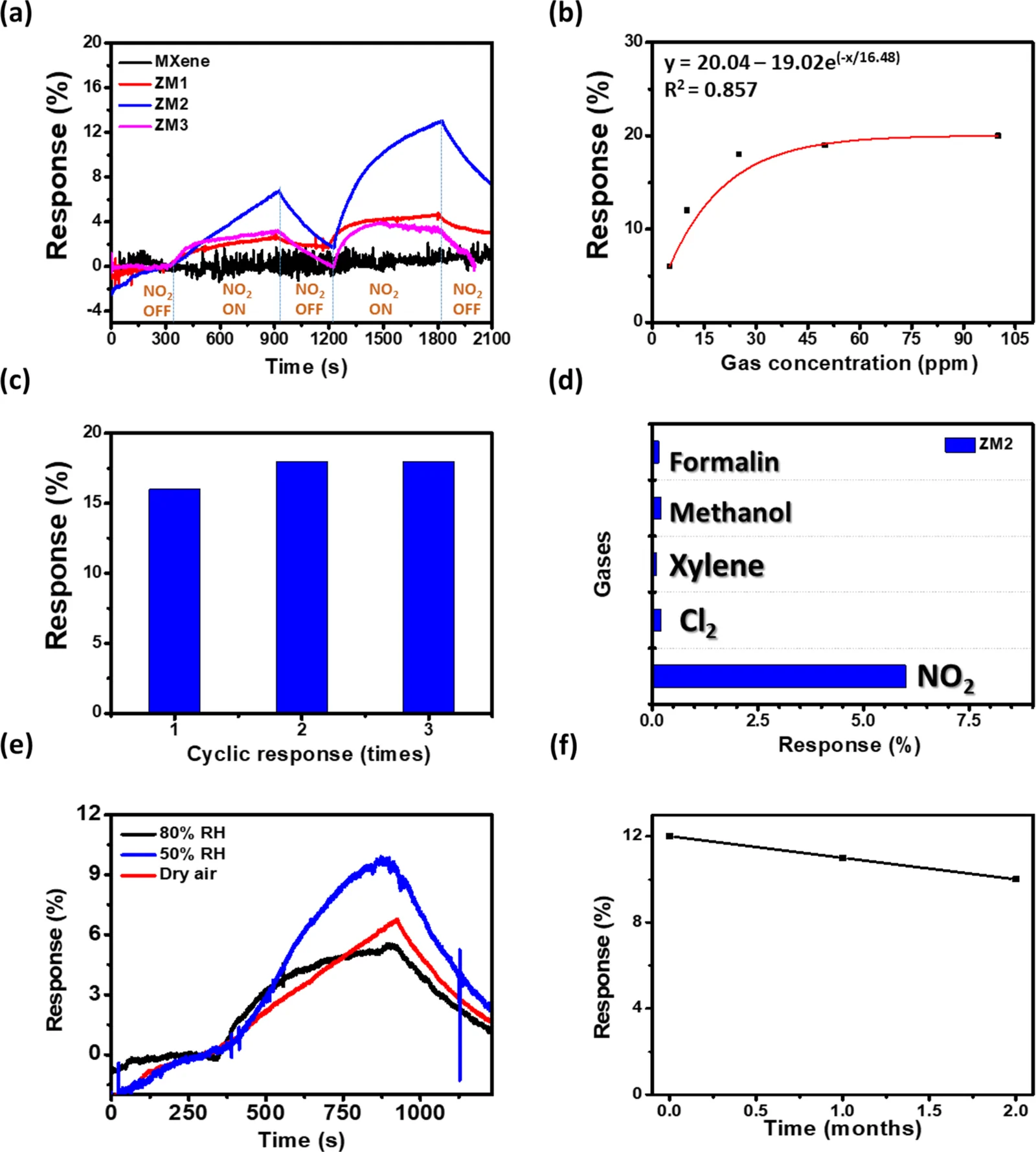
Gas sensing performance of (a) synthesized samples toward NO_2_ (5–10 ppm), (b) response value of ZM2 at different concentrations (5–100 ppm), (c) stability test of ZM2 toward NO_2_ (25 ppm), (d) selectivity properties of ZM2 sensor using different gases (5 ppm). Response value of ZM2 at (e) different relative humidity of NO_2_ (5 ppm) and (f) after 2 months of storage toward NO_2_ (10 ppm).

Gas sensing measurements were carried out using the as-prepared samples. Figure 6a depicts the response and recovery value of pure Ti_3_C_2_T*_x_* and ZnO/Ti_3_C_2_T*_x_* sensors to 5 and 10 ppm NO_2_ at room temperature. No response was obtained for pure MXene, ascribing its metallic properties. In contrast, the ZnO/Ti_3_C_2_T*_x_* sensor revealed a higher response to NO_2_ in comparison with the pure Ti_3_C_2_T*_x_* sensor under the same conditions. The response value of ZM1, ZM2, and ZM3 exposed to concentrations of 5 ppm (10 ppm) NO_2_ were calculated around 2.5 (4.5%), 6.2 (12.1%), and 3.1 (3.6 %), respectively. ZM2 was identified as the optimal ratio for sensor performance and thus selected for subsequent testing. The NO_2_ sensing ability of pristine ZnO was difficult to assess due to its large resistance at room temperature. ZM2 was exposed to various concentrations of NO_2_ at room temperature, and the response value of ZM2 increased with the increase of NO_2_ concentration from 5 to 100 ppm. The sensor response increased progressively with increasing NO_2_ concentration and exhibited an approximately linear trend (*R*^2^ = 0.857). A slight deviation from linearity was observed at NO_2_ concentration above 50 ppm, suggesting the onset of response saturation (Figure 6b).

Figure 6c depicts the three-cycle test for the optimized ZnO/Ti_3_C_2_T*_x_* sensor (ZM2) to 25 ppm NO_2_, demonstrating good reproducibility without any prominent change. Moreover, the selectivity of ZM2 sensor to different gases was conducted for various gases, including NO_2_, Cl_2_, xylene, methanol and formalin, under similar conditions (5 ppm). As shown in Figure 6d, the ZM2 sensor exhibited high selectivity for NO_2_ molecules compared to other gases.

Relative humidity (RH) plays a critical role in gas sensing performance. Therefore, the response of the ZM2 composite under varying humidity levels was evaluated, as illustrated in Figure 6e. The ZM2 sensor exhibited the highest response at 50% RH for 5 ppm NO_2_. At low RH, the excellent humidity tolerance of the composite is attributed to its functional groups, which form weak hydrogen bonds with H_2_O molecules and provide –OH and –O groups that facilitate electron transport through the inherent hydrophilicity of the surface functional groups. As RH increases, the response value gradually decreases. This decline is ascribed to the physisorption of H_2_O molecules on the sensor surface, which hinders NO_2_ adsorption. Moreover, the interaction between H_2_O and NO_2_ molecules impedes the direct interaction between chemisorbed oxygen species and the target gas, thereby modulating ionic conduction within the sensing material and ultimately reducing the gas response. As shown in Figure 6f, the sample exhibits a slight decrease in sensing performance after two months of storage. This degradation can be attributed to surface oxidation and the deactivation of active sites on the sample surface.

The sensor response performance of previously reported NO_2_ sensors is compared in Table 1. Although the ZM2 sensor developed in this study does not exhibit the highest response, it remains a promising candidate for NO_2_ detection at room temperature.

**Table 1:** NO_2_ sensor performance of MXene-based composites.

Composites	Response	Gas concentration	Operation temperature	Ref.

ZnO MOF/Ti_3_C_2_T*_x_*	46.3%	500 ppb	180 °C	[45]
CuO/TiO_2_/Ti_3_C_2_T*_x_*	25%	100 ppm	UV irradiation	[46]
CuO/Ti_3_C_2_T*_x_*	57%	100 ppm	RT	[47]
CuO/TiO_2_/Ti_3_C_2_T*_x_*	15.3%	10 ppm	RT	[48]
Cr_2_O_3_/TiO_2_/Ti_3_C_2_T*_x_*	no response	10 ppm	RT	[49]
N doped Ti_3_C_2_T*_x_*	1.5%	100 ppm	RT	[50]
In_2_O_3_/Ti_3_C_2_T*_x_*	4%	5 ppm	RT	[51]
SnO_2_/Nb_2_CT*_x_*	13.5%	100 ppm	RT	[52]
SnS_2_/Ti_3_C_2_T*_x_*	5.9%	10 ppm	RT	[53]
ZnO/Ti_3_C_2_T*_x_*	12.1%	10 ppm	RT	this work

### Plausible mechanism

The ZnO/Ti_3_C_2_T*_x_* composite exhibited enhanced NO_2_ sensing performance in comparison with pure ZnO and Ti_3_C_2_T*_x_* MXene. Since the pristine Ti_3_C_2_T*_x_* sensor displayed a negligible response toward NO_2_, the enhanced sensing performance of the heterostructures can be ascribed to the synergistic interfacial interactions of components. First, Ti_3_C_2_T*_x_* possesses a higher work function (4.2 eV) than ZnO (3.8 eV) [54], so electrons spontaneously flow from ZnO to Ti_3_C_2_T*_x_* MXene site until Fermi level equilibrium is established. This charge redistribution induces upward band bending in ZnO and leading to the formation of an interfacial electron depletion layer at the ZnO/Ti_3_C_2_T*_x_* interface.

When the ZnO/Ti_3_C_2_T*_x_* composites were exposed to synthetic air, oxygen molecules were chemisorbed on the sensor surface. As a result, O_2_ molecules could trap the free electrons from the ZnO/Ti_3_C_2_T*_x_* to create a series of oxygen ionic species (O_2_^−^ and O^2−^) [55]. In synthetic air, the extraction of electrons further widens the electron depletion layer, resulting in an increase in the electrical resistance of sensing layer.

**Figure 7:**
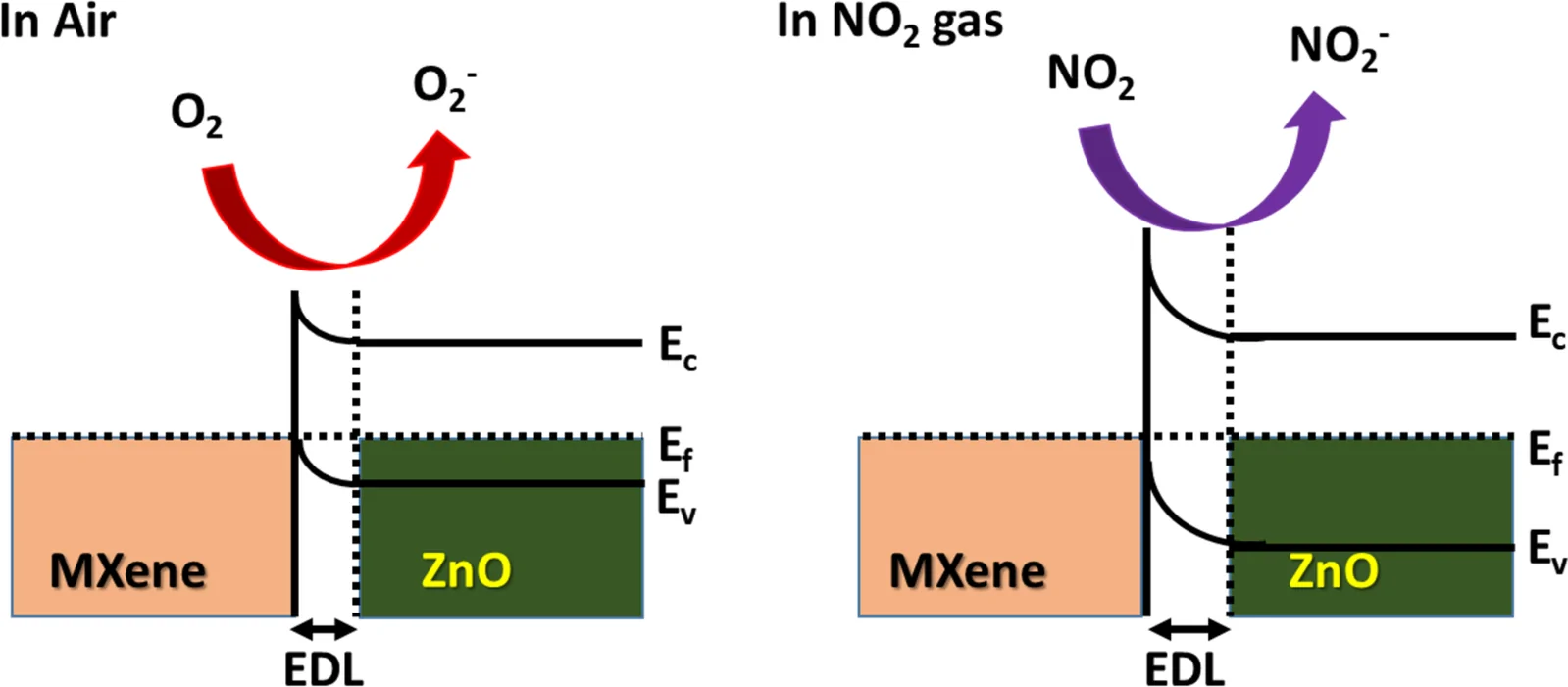
Band structures of ZnO/Ti_3_C_2_T*_x_* heterostructure in air and in NO_2_.

NO_2_ acts as strong electron acceptor; the NO_2_ molecules tend to react with adsorbed oxygen and withdraw electrons, resulting in the electron depletion layer becoming wider, upward band bending in ZnO, and an increase in the sensor’s resistance (Figure 7). In addition, the oxygen vacancies of ZnO could facilitate the adsorption process, which supports oxygen adsorption and facilitates charge transfer. Moreover, the functional groups of MXene (–O, –OH, –F) offer abundant favorable sites for NO_2_ through hydrogen bonding, thereby facilitating NO_2_ adsorption and interfacial charge transfer [56]. The adsorbed NO_2_ molecules further withdraw electrons from MXene, contributing to the enlargement of the electron depletion layer of the sensor layer [45,57]. The gas sensing reactions can be summarized as follows:


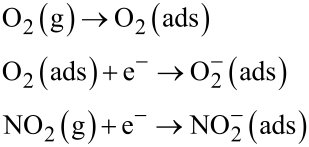


## Conclusion

In summary, we developed a synthesis method for the facile preparation of a ZnO/Ti_3_C_2_T*_x_* composite with a lamellar structure for gas sensing. The enhanced sensitivity and stability of the NO_2_ sensors are attributed to an increased electron density transfer between NO_2_ molecules and the sensing layers. The underlying sensing mechanism responsible for the observed improvements in NO_2_ sensing performance was systematically examined, providing valuable insights into the complex interactions between the composite materials and NO_2_ molecules. This understanding of the sensing mechanism offers guidance for the design and optimization of future MXene-based gas sensors, promoting the development of highly efficient and reliable platforms for environmental monitoring and industrial applications.

## Experimental

### Synthesis of pure Ti_3_C_2_T*_x_* MXene and ZnO/Ti_3_C_2_T*_x_* nanocomposites

Typically, 0.4 g of LiF (Sigma) was dissolved in 5 mL of 9 M HCl (China chem, 36 wt %) aqueous solution under vigorous stirring for 30 min. Then, a certain amount of Ti_3_AlC_2_ MAX phase (100 mesh, purchased from Sigma) was slowly added into the mixture solution and kept at 50 °C in an oil bath. After one day, the reaction solution was filtered and washed using vacuum filtration and centrifuged at 3500 rpm with DI water until pH ≈ 7.0. Finally, the Ti_3_C_2_T*_x_* MXene was collected and freeze-dried for further fabrication.

**Figure 8:**
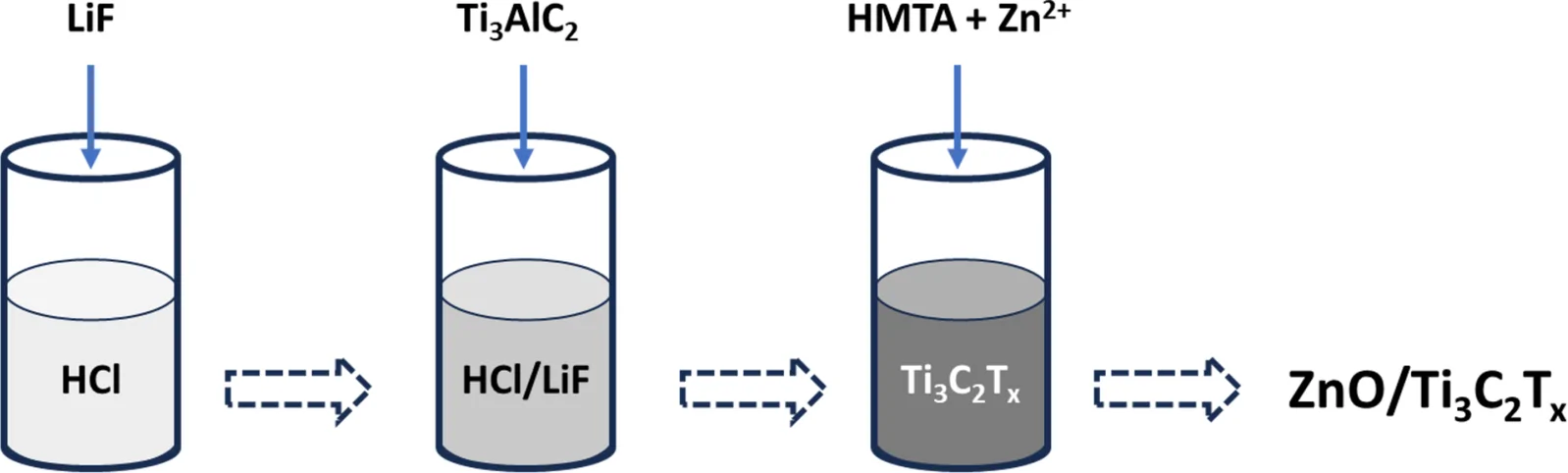
Schematic illustration of the synthesis of ZnO/Ti_3_C_2_T*_x_* composites.

The ZnO/Ti_3_C_2_T*_x_* heterojunction was prepared via a facile self-assembly process, as displayed in Figure 8. In brief, a certain amount of Ti_3_C_2_T*_x_* MXene (10, 20, and 30 mg) was well dispersed in 50 mL of deionized water by ultrasonication. Subsequently, 0.744 g of zinc nitrate hexahydrate (Zn(NO_3_)_2_·6H_2_O) was added to the Ti_3_C_2_T*_x_* MXene suspension under vigorous magnetic stirring for 30 min to ensure complete dissolution and homogeneous mixing. Then, 0.350 g of hexamethylenetetramine (HMTA, C_6_H_12_N_4_) was added to the mixture solution under continuous stirring. The resulting suspension was maintained in an oil bath at 70 °C for 2 h. After completion of the reaction, the precipitate was collected and washed several times with DI water to remove residual reactants and by-products. Subsequently, the obtained product was aged at 100 °C under a nitrogen atmosphere to facilitate the formation of a stable ZnO/Ti_3_C_2_T*_x_* interface while minimizing the oxidation of Ti_3_C_2_T*_x_* during the post-synthesis process. The composites prepared with 10, 20, and 30 mg of Ti_3_C_2_T*_x_* MXene were designated as ZM1, ZM2, and ZM3, respectively. For comparison, pure ZnO nanostructures were synthesized following the same procedure without the addition of Ti_3_C_2_T*_x_* MXene.

### Materials characterization

The morphology and elemental analysis of the selected samples were recorded by scanning electron microscopy (SEM, JEOL, IT200 Japan). The phase structure of samples was obtained by an X-ray diffractometer (XRD, D8 Advance Eco, Bruker AXS Germany) with 2θ ranging from 5° to 70° and Cu Kα irradiation (λ = 0.154056 nm). The porous structure was analyzed by nitrogen adsorption/desorption isothermals using the Brunner–Emmett–Teller method (BET, TriStar II Plus 3030, Micromeritics USA).

### Gas sensing measurements

The gas sensors were composed of a glass substrate and a thin layer of as-synthesized composites. In brief, the precise amounts of prepared samples were first dispersed in ethanol and well-sonicated for 15 min. Then, 5 μL dispersion was drop-cast onto the cleaned glass substrate. Finally, the sensors underwent aging in a vacuum furnace to remove ethanol and improve the bonding between the substrate and gas sensing material.

For gas sensing measurements, the substrate was located in a stainless steel chamber. The signal was analyzed using a source meter instrument (Keithley 2400). The gas flow rate was set to 500 sccm and adjusted by mass flow controllers. The ratio of synthetic air to target gas was changed to obtain the desired gas concentration. Moreover, the sensor response is defined as *S* = (*R*_g_/*R*_a_ − 1) × 100%, where *R*_a_ is the resistance under synthetic air after stabilization, and *R*_g_ is the resistance under target gas. The response/recovery time is calculated as the time taken for composites to achieve 90% of the maximum resistance change.

## Data Availability

Data generated and analyzed during this study is available from the corresponding author upon reasonable request.
